# Association between disorganization of retinal inner layers and visual acuity after proliferative diabetic retinopathy surgery

**DOI:** 10.1038/s41598-019-48679-z

**Published:** 2019-08-22

**Authors:** Tomoyuki Ishibashi, Susumu Sakimoto, Nobuhiko Shiraki, Kentaro Nishida, Hirokazu Sakaguchi, Kohji Nishida

**Affiliations:** Department of Ophthalmology, Osaka University Graduate School of Medicine, Suita, Japan

**Keywords:** Prognostic markers, Retinal diseases

## Abstract

We report to evaluate if disorganization of the retinal inner layers (DRIL) obtained by swept-source optical coherence tomography (SS-OCT) predicts the postoperative best-corrected visual acuity (BCVA) to treat proliferative diabetic retinopathy (PDR). Twenty-one eyes of 21 patients who underwent vitrectomy for PDR were studied retrospectively. BCVA and SS-OCT images were obtained until 6 months postoperatively. The associations between BCVA and SS-OCT parameters measured in a 1-mm central foveal area were evaluated. The DRIL length, external limiting membrane disruption, and ellipsoid zone (EZ) disruption 1 month postoperatively were associated positively with the postoperative logarithm of the minimum angle of resolution (logMAR) BCVA at 1, 3, and 6 months (1 month, p = 0.009, p = 0.013, p = 0.001; 3 months, p = 0.03, p = 0.021, p = 0.002; and 6 months, p = 0.021, p = 0.013, and p = 0.005, respectively). The eyes with a 500-µm or longer DRIL 1 month postoperatively (19%, 4/21 eyes) had significantly worse VA at 1, 3, and 6 months postoperatively (p = 0.007, p = 0.008, and p = 0.020, respectively). Multilinear regression analysis of all visits until 6 months postoperatively showed that the DRIL was correlated more significantly (p = 0.0004) with logMAR BCVA than the disrupted EZ length. The DRIL in the early postoperative period may predict the visual outcomes after treating PDR.

## Introduction

Diabetic retinopathy (DR) is the leading cause of blindness among working-age patients, and the prevalence of diabetes mellitus is predicted to increase globally^1,2^. Proliferative diabetic retinopathy (PDR) is the advanced stage of DR characterized by preretinal neovascularization^3^, which causes vitreous hemorrhages and traction retinal detachments. Those complications are possible indications for pars plana vitrectomy (PPV), but the visual prognosis in some cases is poor. Numerous studies have reported the visual outcome after diabetic vitrectomy; however, the factors predictive of the visual outcomes remain to be elucidated^4^. The identification of reliable biomarkers that predict the visual acuity (VA) after diabetic vitrectomy is important for the care of patients with PDR.

Sun and associates first characterized the disorganization of the retinal inner layers (DRIL) as the inability to distinguish any of the boundaries of the ganglion cell layer-inner plexiform layer (GCL-IPL) complex, inner nuclear layer (INL), and outer plexiform layer (OPL) in the horizontal B-scan of optical coherence tomography (OCT) images^5,6^. Regarding diabetic macular edema (DME), those investigators reported that the DRIL was associated with worse VA and that the changes in the DRIL predicted the subsequent changes in VA. The purpose of the current study was to evaluate the correlation between the DRIL and postoperative VA in eyes with PDR that required vitrectomy. Because one of the most frequent complications of PDR is vitreous hemorrhage, which prevents retinal evaluation, we used swept-source OCT (SS-OCT) to analyze patients during the first month postoperatively. SS-OCT uses a longer wavelength (1,050 nm) than SD-OCT and is expected to penetrate through opacities or into the deeper tissues and obtain sharp images in the early postoperative period.

## Results

Twenty-one eyes of 21 patients (14 men, 7 women; mean age, 62.4 ± 13.0 years; range 41–82 years) who underwent PPV with cataract surgery and a SS-OCT examination were included in this study. All eyes were diagnosed with PDR without any other retinal diseases preoperatively. Table 1 shows the preoperative characteristics of the patients and OCT parameters 1 month postoperatively. The surgical indications included vitreous hemorrhage (12 patients, 57%), traction retinal detachment (5 patients, 24%), and epiretinal membrane (4 patients, 19%). One month postoperatively, seven eyes (33%) had cystoid macular edema (CME) and none had subretinal fluid (SRF). Twenty-one (100%) eyes had hyperreflective foci (HRF); one (5%) eye had HRF only in the inner retina and 20 eyes (95%) had HRF in the inner and outer retina. The mean central subfield thickness (CST) was 289 ± 111 µm (range, 100–633 µm). The mean size of the external limiting membrane (ELM) disruption was 109 ± 187 µm (range, 0–551 µm), and the mean size of the ellipsoid zone (EZ) disruption was 434 ± 379 µm (range, 0–1,000 µm). The total area of CME was 0.11 ± 0.36 mm^2^ (range, 0–1.67 mm^2^). Four (19%) eyes were with a mean DRIL length of 500 µm or longer. The mean DRIL length of all B-scans was 350 ± 234 µm (range, 0–1,000 µm). The percentage of the scans with DRIL out of five scans in each eye was 30 ± 32% (range, 0–100%).

**Table 1:** Baseline patient characteristics and OCT parameters 1 month postoperatively.

Parameter	Values
Age (years) (mean ± SD; range)	62.4 ± 13.0; 41–82
Gender, n (%)	
Male	14 (67)
Female	7 (33)
Main surgical indication, n (%)	
VH	12 (57)
TRD	5 (24)
ERM	4 (19)
OCT parameters 1 month postoperatively	
Mean DRIL ≥500 μm, n (%)	4 (19)
CME, n (%)	7 (33)
SRF, n (%)	0 (0)
HRF (inner retina), n (%)	1 (5)
HRF (inner + outer retina), n (%)	20 (95)
Mean CST (μm) (mean ± SD; range)	289 ± 111; 100–633
Mean DRIL length (μm) (mean ± SD; range)	350 ± 234; 0–1000
Proportion of scans with DRIL out of 5 scans (%) (mean ± SD; range)	30 ± 32; 0–100
Mean ELM disruption (μm) (mean ± SD; range)	109 ± 187; 0–551
Mean EZ disruption (μm) (mean ± SD; range)	434 ± 379; 0–1000
Total area of CME (mm^2^) (mean ± SD; range)	0.11 ± 0.36; 0–1.67

OCT, optical coherence tomography; SD, standard deviation; VH, vitreous hemorrhage; TRD, traction retinal detachment; ERM, epiretinal membrane; DRIL, disorganization of retinal inner layers; CME, cystoid macular edema; SRF, subretinal fluid; HRF, hyperreflective foci; CST, central subfield thickness; ELM, external limiting membrane; EZ, ellipsoid zone.

Table 2 shows the associations between the OCT parameters 1 month postoperatively and the postoperative logarithm of the minimal angle of resolution (logMAR) best corrected visual acuity (BCVA) at 1, 3, and 6 months postoperatively. No significant correlations was seen between the CST at 1 month and the postoperative logMAR BCVA at each visit (1 month, p = 0.39; 3 months, p = 0.16; and 6 months, p = 0.40) or between the total area of the CME at 1 month and the postoperative logMAR BCVA at each visit (1 month, p = 0.40; 3 months, p = 0.41; and 6 months, p = 0.49). The mean DRIL length at 1 month was correlated positively with the postoperative logMAR BCVA at each visit (1 month, p = 0.009; 3 months, p = 0.030; and 6 months, p = 0.021). The percentages of the five scans with DRIL in each eye at 1 month were correlated positively with the postoperative logMAR BCVA at each visit (1 month, p = 0.002; 3 months, p = 0.009; and 6 months, p = 0.008). The mean length of the ELM disruption at 1 month was correlated positively with the postoperative logMAR BCVA at each visit (1 month, p = 0.013; 3 months, p = 0.021; and 6 months, p = 0.013). The mean length of the EZ disruption at 1 month was correlated positively with the postoperative logMAR BCVA at each visit (1 month, p = 0.001; 3 months, p = 0.002; and 6 months p = 0.005).

**Table 2:** Simple linear regression analysis between OCT parameters 1 month postoperative and postoperative logMAR BCVA.

	LogMAR BCVA at 1 month			LogMAR BCVA at 3 month			LogMAR BCVA at 6 month		
	Regression coefficient	R^2^	p value	Regression coefficient	R^2^	p value	Regression coefficient	R^2^	p value
Mean CST	0.001	0.04	0.39	0.002	0.14	0.16	0.001	0.05	0.40
Mean DRIL length	0.001	0.31	0.009	0.001	0.29	0.030	0.001	0.32	0.021
Percentage of the scans with DRIL ≥500 μm out of 5 scans	1.113	0.39	0.002	1.001	0.40	0.009	1.078	0.40	0.008
Mean ELM disruption	0.002	0.29	0.013	0.002	0.33	0.021	0.002	0.36	0.013
Mean EZ disruption	0.001	0.44	0.001	0.001	0.49	0.002	0.001	0.44	0.005
Total area of CME	0.307	0.04	0.40	0.297	0.05	0.41	0.275	0.03	0.49

OCT, optical coherence tomography; logMAR, logarithm of the minimum angle of resolution; BCVA, best-corrected visual acuity; CST, central subfield thickness; DRIL, disorganization of retinal inner layers; ELM, external limiting membrane; EZ, ellipsoid zone; CME, cystoid macular edema.

We divided the patients into two groups: those with a mean DRIL length less than 500 µm 1 month postoperatively (DRIL−) and those with a mean DRIL length of 500 µm or longer 1 month postoperatively (DRIL+). The postoperative BCVA was significantly better in the DRIL− group than in the DRIL+ group at each visit (1 month, p = 0.007; 3 months p = 0.008; and 6 months p = 0.020) (Table 3).

**Table 3:** Comparison of postoperative visual acuity between DRIL− and DRIL+.

	*DRIL−	^†^DRIL+	p value
LogMAR BCVA 1 month postoperatively (median [IQR])	0.3 [0.2–0.5]	1.1 [1–1.5]	0.007
LogMAR BCVA 3 month postoperatively (median [IQR])	0.3 [0.2–0.5]	1.0 [0.9–1.4]	0.008
LogMAR BCVA 6 month postoperatively (median [IQR])	0.3 [0.1–0.5]	1.0 [0.9–1.5]	0.02

DRIL, disorganization of retinal inner layers; logMAR, logarithm of the minimum angle of resolution.

BCVA, best-corrected visual acuity; IQR, interquartile range.

^*^DRIL− group includes eyes with a mean DRIL length <500 µm 1 month postoperatively.

^†^DRIL+ group includes eyes with mean DRIL length ≥500 µm 1 month postoperatively.

Multilinear regression was performed to predict the VA based on the length of the DRIL length and EZ disruption across all visits up to 6 months postoperatively (Table 4). The mean length of the DRIL was a significant (p = 0.0004) predictor of the BCVA, whereas the mean length of the EZ disruption was not associated significantly (p = 0.45) with the BCVA.

**Table 4:** Multilinear regression analysis of logMAR BCVA with lengths of DRIL and EZ disruption across all visits.

	Regression coefficient	p value	95% confidence interval (lower)	95% upper confidence interval
Mean DRIL length	0.001	0.0004	0.0006	0.002
Mean EZ disruption length	0.0002	0.45	−0.00036	0.0008

LogMAR, logarithm of the minimum angle of resolution; BCVA, best-corrected visual acuity; DRIL, disorganization of retinal inner layers; EZ, ellipsoid zone.

## Discussion

The current study evaluated the association between SS-OCT parameters and the postoperative BCVA in PDR. The horizontal and vertical extents of the DRIL, ELM disruption, and EZ disruption 1 month postoperatively were correlated negatively with the logMAR BCVA at 1, 3, and 6 months postoperatively. The eyes with a DRIL 500 µm or longer 1 month postoperatively were associated significantly with worse BCVA at 1, 3, and 6 months postoperatively compared to eyes with a DRIL less than 500 µm long. Multilinear regression analysis showed that the length of the DRIL was correlated significantly with the BCVA at all visits until 6 months postoperatively. These results suggested that the DRIL 1 month postoperatively may predict the visual outcomes of surgeries performed to treat PDR and that the DRIL may reflect the VA at the same time. To the best of our knowledge, this is the first study to investigate the association between the DRIL and VA after surgery to treat PDR using SS-OCT.

Both the reports on PDR after surgery and numerous studies of various macular diseases have reported that disruption of the outer retina is a significant parameter to predict the visual prognosis^7–11^. In the current study, simple linear regression analysis, the presence or length of the DRIL, and the integrity of the ELM and EZ were considered as the factors that might predict the visual outcomes; however, multilinear regression analysis showed that the DRIL, but not the EZ, was associated with the visual outcomes. Radwan *et al*. reported previously that the length of the DRIL was associated significantly with the VA after resolution of center-involved DME, whereas the disrupted lengths of the ELM and EZ were not associated significantly^12^. The reason that the inner retina was more impaired compared to the outer retina is unclear. Several studies have suggested that the inner retinal status was more important for assessing the visual prognosis in eyes with an ERM^13–15^. Govetto *et al*. reported that the presence of ectopic inner foveal layers in ERMs is a newly identified factor associated with significant visual loss^14^. Cho *et al*. reported that the inner retinal irregularity index was the factor that was most significantly correlated with visual outcomes before and after ERM surgery^13^.

Although the mechanism of development of the DRIL is unknown, previous studies have reported that the DRIL was identified as a potential biomarker for macular edema caused by DR, retinal vein occlusion, and uveitis^6,16–18^. The diseases that involve retinal vascular dysfunction can disrupt the blood retinal barrier, which is followed subsequently by accumulation of extracellular fluid in the intra- or subretinal space. The DRIL obviously is a result of destruction of the neural structure due to compromised retinal microcirculation; however, PDR also includes a fibrovascular membrane that creates tractional stress on the macula. Zur *et al*. reported recently that the DRIL also can be a reliable biomarker for eyes that underwent surgery for ERM^15^. Thus, the combined mechanisms that include vascular and mechanical stress-related factors might have exaggerated formation of DRIL in eyes with PDR.

The inability to visualize the boundaries of the inner retinal layers may indicate anatomic collapse in the visual transmission pathway. Because the inner retinal layers include the bipolar, amacrine, or horizontal cells, which correspond to the second neuron in the visual system, this may indicate that the DRIL disrupts pathways that transmit visual information from the photoreceptors to the ganglion cells. These anatomic alterations also might be responsible for less robust associations observed with other outer retinal parameters observed by OCT^5^.

The current study had several limitations. This study was retrospective and the sample size was relatively small. As mentioned previously, the reason that the DRIL and not the EZ was identified as a crucial factor for visual outcomes is unknown; however, the current sample size might have affected this. On the other hand, the sample size were still appropriate because of following reason. First, because the current study has a pilot nature, we were not able to hypothesize a difference to be detected in the analysis. Second, for example, outcomes comparing visual acuity between DRIL− and DRIL+ which are shown in Table 3 demonstrated median logMAR approximately 0.3 and 1.0, respectively. Those values still seem to be appropriate and the differeces were reasonable when we see PDR patients who received surgery. That strengthens the fact that as long as we performed appropriate statistical analysis, the data we detected in the current study were significant. Another limitation of the current study is that there is controversy regarding the inter-observer difference in DRIL evaluation^19^. To evaluate DRIL objectively, an automated detection system of DRIL should be developed in future^20,21^. Nonetheless, the horizontal and vertical extents of the DRIL 1 month postoperatively were correlated negatively with the logMAR BCVA postoperatively. Moreover, the length of the DRIL was correlated significantly with the BCVA at all visits until 6 months postoperatively. These results demonstrated that the DRIL evaluated 1 month postoperatively may be a prognostic factor that present the visual outcomes after PDR surgeries. The detailed definition of DRIL and the technology to detect DRIL automatically is required for future application in the clinic where the visual prognosis can be predicted during the early postoperative stage in eyes with PDR.

## Methods

This single-site, retrospective study adhered to the tenets of the Declaration of Helsinki; the institutional review board of Osaka University Hospital approved the study. Informed consent was obtained from all individual participants. We enrolled 21 eyes of 21 consecutive patients who had undergone both 25-gauge PPV for PDR from May 2015 to March 2018 and an SS-OCT examination at the Department of Ophthalmology of Osaka University Hospital. The inclusion criteria were eyes with PDR and an SS-OCT examination 1 month postoperatively. Eyes were excluded that had any other retinal diseases, a history of other intraocular surgeries, postoperative poor-quality OCT images, or an additional surgery within 6 months after the initial surgery.

PPV was performed using a 25-gauge vitrectomy system (Constellation Vision System; Alcon Laboratories, Inc., Fort Worth, Texas, USA) and a wide-angle viewing system (Resight, Carl Zeiss Meditec, Oberkochen, Germany). Phacoemulsification and intraocular lens implantation were performed simultaneously in all phakic eyes. A core vitrectomy was performed with intravitreal injection of triamcinolone acetonide to visualize the vitreous gel. A peripheral vitrectomy was performed under scleral indentation. Fibrovascular membranes were removed with a vitreous cutter, vitreoretinal forceps, and scissors. Panretinal photocoagulation was applied up to the vitreous base under scleral indentation in all eyes. Internal limiting membrane peeling was performed at the surgeons’ discretion. Fluid-gas exchange was performed in eyes with retinal breaks or a rhegmatogenous retinal detachment. Air or 20% sulfur hexafluoride was used in these cases.

All patients underwent comprehensive ophthalmic examinations at all visits until 6 months postoperatively. The examinations included measurements of BCVA and intraocular pressure, slit-lamp biomicroscopy, fundus photography, and SS-OCT (DRI-OCT, Topcon Medical Systems, Tokyo, Japan). This SS-OCT system operates at a speed of 100,000 A-scans/second using a 1,050-nm wavelength light source and has an 8-µm axial resolution and 20-µm lateral resolution. For each eye, we used the 5 Line Cross mode with a 12-mm scan length and 0.1-mm spacing to acquire five horizontal B-scan images centered on the fovea.

### Image analysis

For each study eye, a 1-mm overlay centered on the fovea was placed over each of the five horizontal scans. We investigated the following in this area as previously reported^18^: the presence of CME, SRF, and HRF; CST (µm); DRIL length (µm); vertical extent of the DRIL (defined as the number of scans out of five with DRIL calculated as a percentage); ELM disruption (µm); EZ disruption (µm); and total area of the CME (mm^2^). ImageJ software (National Institutes of Health, Bethesda, Maryland, USA) was used to manually measure each OCT parameter. The means of the CST, DRIL, ELM disruption, and EZ disruption were calculated from the five horizontal scans at each visit.

### Definition of DRIL

**Figure 1:**
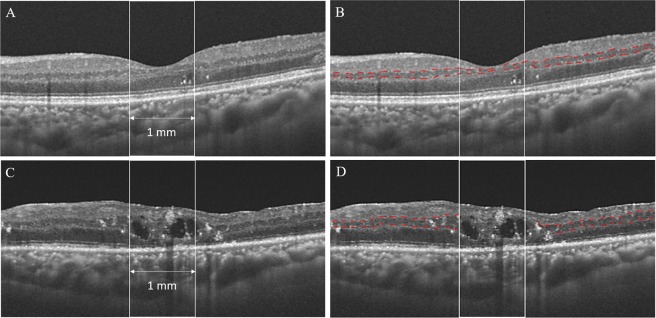
Disorganization of retinal inner layers (DRIL) is evaluated within 1 mm centered on the fovea. (**A**,**B**) A swept-source optical coherence tomography (SS-OCT) image of a 64-year-old woman whose decimal visual acuity (VA) is 1.0 1 month postoperatively. The boundaries of the inner nuclear layer (INL) are almost clearly identified despite the presence of cystoid macular edema and hyperreflective foci at the outer nuclear layer. (**C**,**D**) A SS-OCT image of a 56-year-old man whose decimal VA is 0.06 1 month postoperatively. The boundaries of the INL cannot be identified clearly.

DRIL was defined as the inability to distinguish any of the boundaries of the GCL-IPL complex, INL, and OPL within 1 mm centered on the fovea (Fig. 1). The length of the DRIL was measured in five horizontal B-scans, and the measurements were averaged for each eye at each visit. Two masked investigators (TI, SS) independently evaluated the DRIL. When there was disagreement, the evaluation was discussed to reach a consensus.

### Statistical analysis

The BCVA were converted to the logarithm of the minimal angle of resolution (logMAR) for all analyses. Counting fingers and hand motions VAs were converted as previously reported^22^. All statistical analyses were conducted using Statcel4 software (OMS, Saitama, Japan). Simple linear regression and multilinear regression analyses were performed to determine the relationship between the BCVA and OCT parameters. The Mann-Whitney U-test was used for group comparisons. P < 0.05 was considered significant.
